# Supplementary figures and images for: The seasonality of diarrheal pathogens: A retrospective study of seven sites over three years
Source: PLoS Negl Trop Dis. 2019 Aug 15;13(8):e0007211. doi: 10.1371/journal.pntd.0007211 (PMC6711541; doi:10.1371/journal.pntd.0007211)

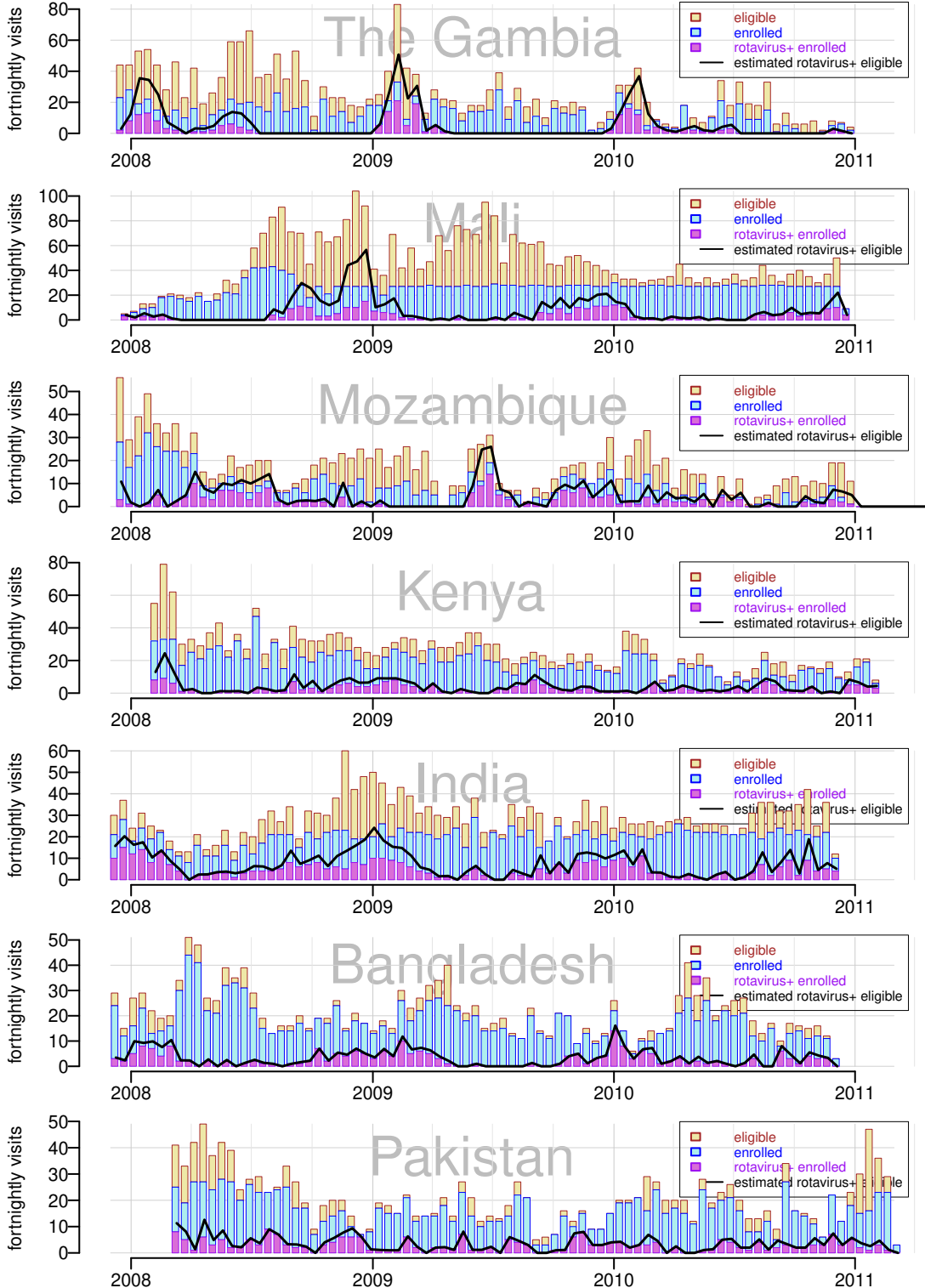

Supplement: S1 Fig — Children with MSD were eligible for the study (gold bars). A sample of them were enrolled in GEMS each fortnight (blue bars) and stools samples were tested for rotavirus and other pathogens. The estimated number of eligible children positive for a rotavirus (black line), is extrapolated from the positivity among enrolled children (purple bars) during the same fortnight. (PDF) [file pntd.0007211.s005.pdf]

# The Gambia

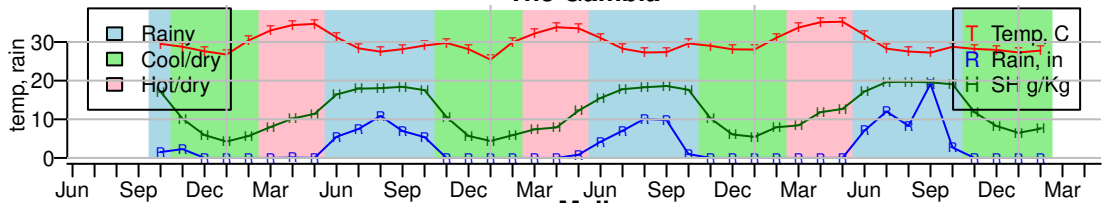

# Mali

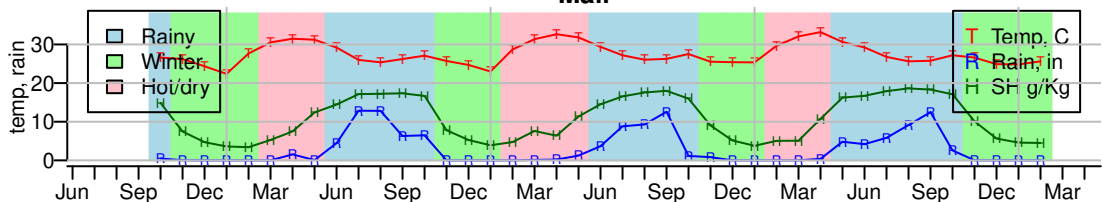

# Kenya

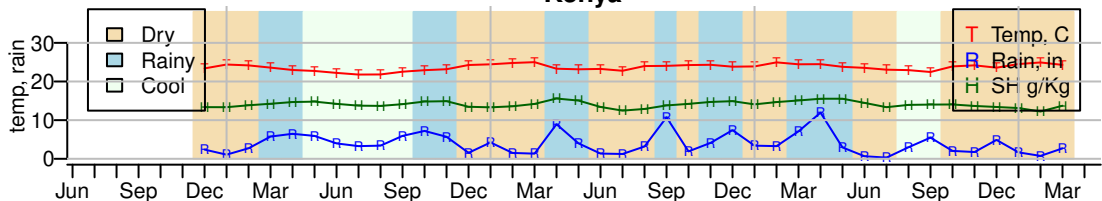

# Mozambique

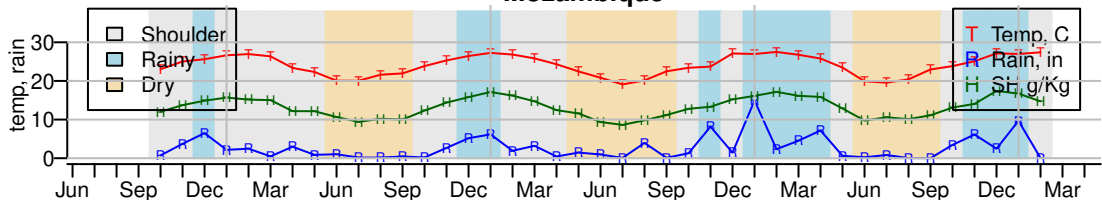

# Pakistan

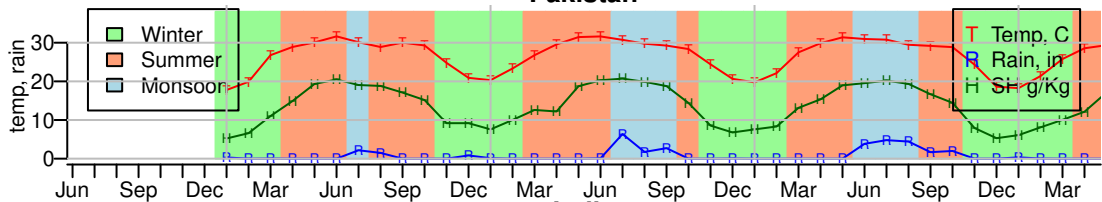

# India

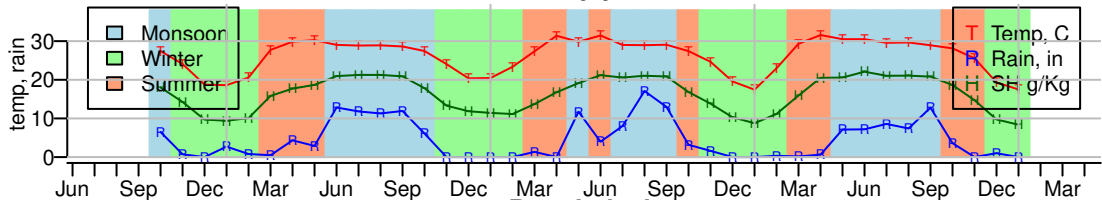

# Bangladesh

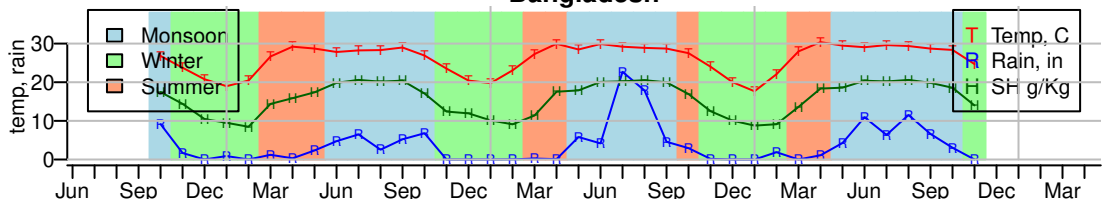

Supplement: S2 Fig — Seasons were defined by a k-means clustering algorithm on PCA-transformed monthly weather covariates. Relative humidity not shown. (PDF) [file pntd.0007211.s006.pdf]

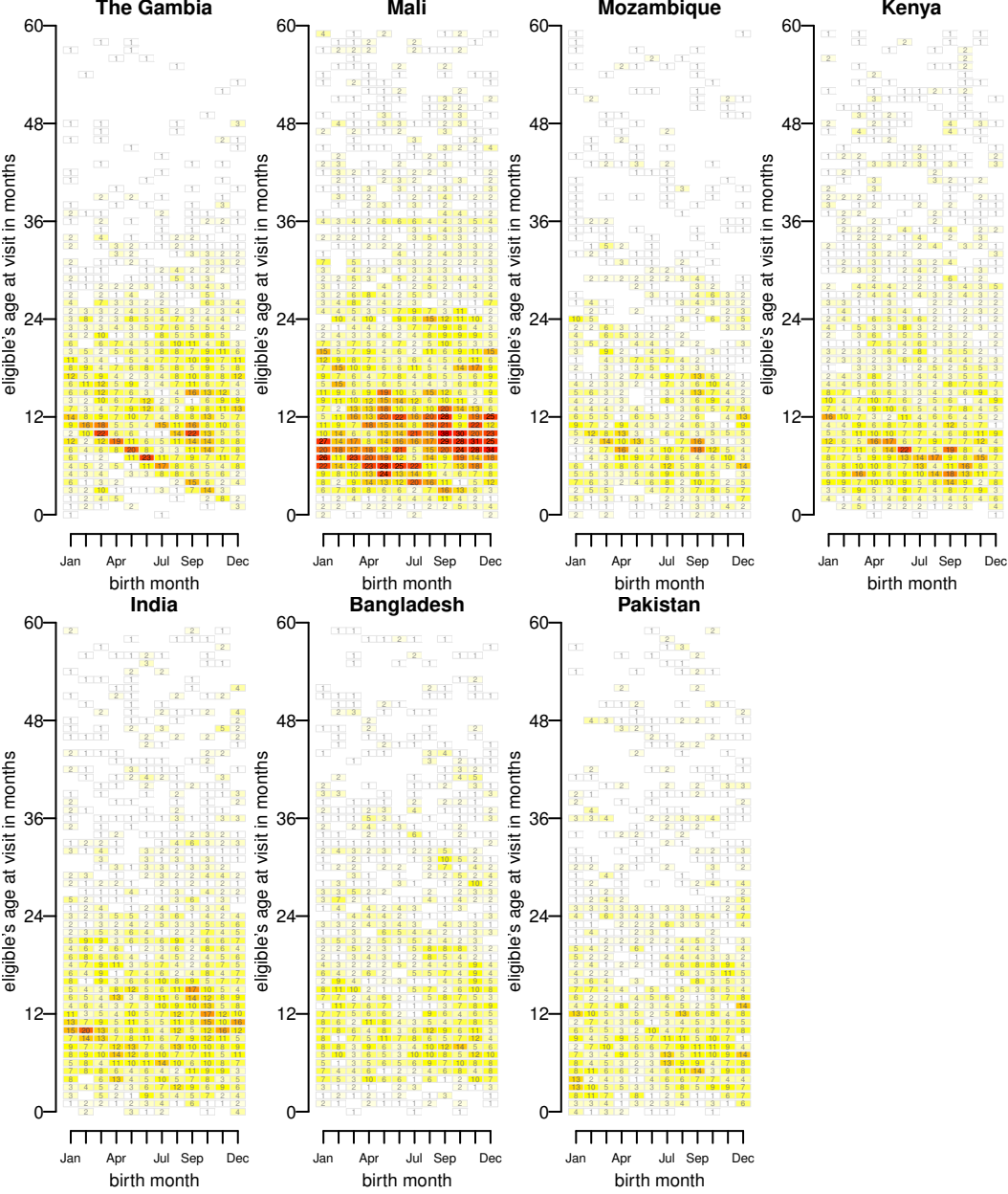

Supplement: S3 Fig — The numbers in the plot are the number of eligible children born in month x and visited the clinic at age y. Yellow areas are low numbers of eligible children, while red numbers are high. Note the diagonal elements in countries where there is a distinct diarrheal disease season. (PDF) [file pntd.0007211.s007.pdf]

**The Gambia**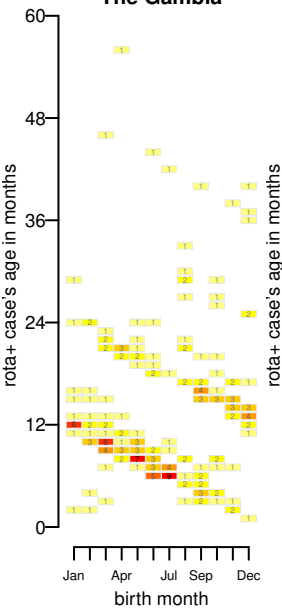**Mali**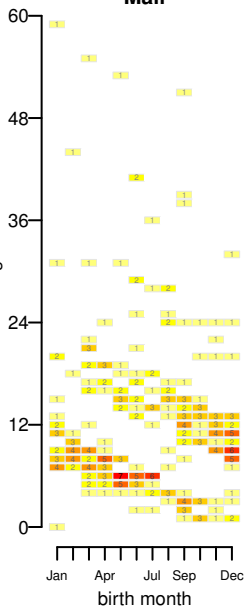**Mozambique**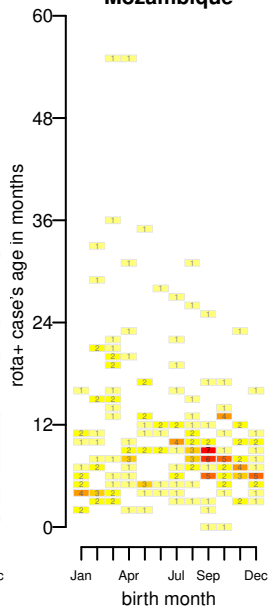**Kenya**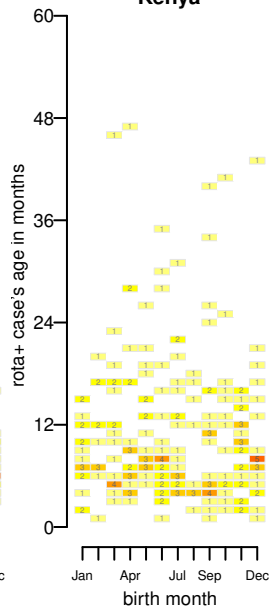**India**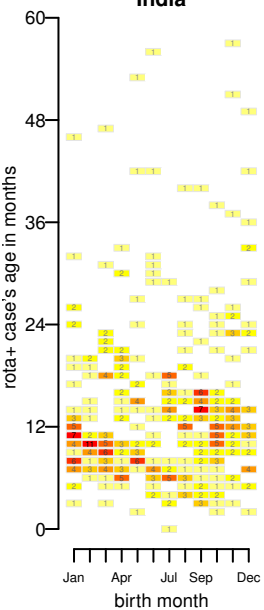**Bangladesh**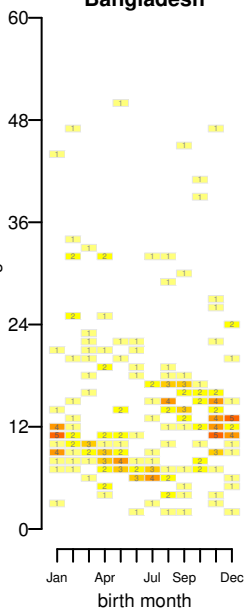**Pakistan**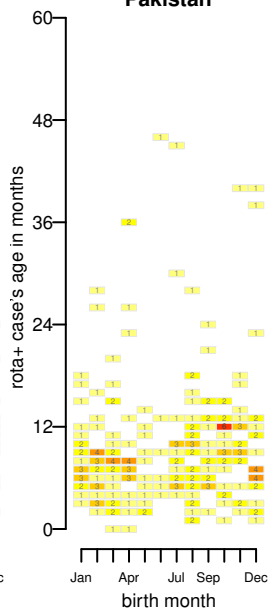

Supplement: S4 Fig — The numbers in the plot are the number of enrolled cases born in month x and were enrolled at age y who were rotavirus-positive. Note the diagonal elements in countries where there is a distinct rotavirus season. Enrollment rates could differ by age group, so one should not compare numbers across age groups (0–11m, 12–23m, 24m and older). (PDF) [file pntd.0007211.s008.pdf]
